# Enzyme-Treated *Zizania latifolia* Extract Protects against Alcohol-Induced Liver Injury by Regulating the NRF2 Pathway

**DOI:** 10.3390/antiox10060960

**Published:** 2021-06-15

**Authors:** Bo Yoon Chang, Hyung Joong Kim, Tae Young Kim, Sung Yeon Kim

**Affiliations:** 1 Institute of Pharmaceutical Research and Development, College of Pharmacy, Wonkwang University, Iksan 54538, Jeonbuk, Korea; pama611@naver.com; 2 BTC Corporation #703, Technology Development Center, 705 Haean-ro, Sangnok-gu, Ansan-si 15588, Gyeonggi-do, Korea; hjkim01@btcbio.com (H.J.K.); tykim@btcbio.com (T.Y.K.)

**Keywords:** *Zizania latifolia*, antioxidant, heme oxygenase-1, nuclear factor erythroid-2-related factor 2, hepatoprotective

## Abstract

Binge drinking patterns easily produce a state of oxidative stress that disturbs liver function. Eventually, this leads to alcoholic liver disease. A safe and effective therapy for alcoholic liver disease remains elusive. Enzyme-treated *Z. latifolia* extract (ETZL) was studied as a potential agent for treating alcohol-induced liver disease. In addition, its underlying mechanisms were elucidated. In the binge model, ETZL was pretreated with alcohol (5 g/kg) three times at 12-h intervals. Our results showed that ETZL pretreatment decreased the serum levels of ALT, AST, ALP, and TG. ETZL treatment appeared to prevent an increase in hepatic TG and MDA levels, and there was a decrease in total GSH following alcohol treatment. Histopathological examination showed that lipid droplets were significantly reduced in the ETZL group compared to the control group. ETZL also exhibited radical scavenging activity. It significantly reduced *t*-BHP-induced cytotoxicity and the production of reactive oxygen species (ROS) in HepG2 cells. ETZL also enhanced NRF2 nuclear translocation and increased expression of the downstream target genes HO-1, NQO1, and GCLC as an antioxidant defense. Finally, ETZL treatment significantly reduced cell death. Our study suggests that ETZL ameliorates binge ethanol-induced liver injury by upregulating the antioxidant defense mechanism.

## 1. Introduction

Fatty liver is classified into two groups: (1) alcoholic fatty liver, in which fat is deposited in the liver due to alcohol consumption, and (2) non-alcoholic fatty liver, in which fat is deposited in the liver, even when drinking a small amount or no alcohol at all [1,2,3].

Excessive drinking causes fat accumulation in liver cells, and the metabolites of alcohol (acetaldyhyde) damage those cells. The occurrence of liver disease caused by alcohol varies greatly, and it depends on the individual and their gender, as well as genetic factors and nutritional status. Patients with chronic liver disease caused by the hepatitis C virus, especially those who are female or poorly nourished, can also suffer severe liver damage from small amounts of alcohol [3,4,5].

It is generally safe for men and women to drink 40 g and 20 g of alcohol per day, respectively. However, the ability to decompose alcohol varies greatly from person to person [6]. Fortunately, an alcoholic fatty liver can be restored to its normal function by avoiding alcohol and receiving enough rest and adequate nutrition. However, about 20–30% of patients who continue to drink alcohol develop alcoholic hepatitis, and 10% progress to simple cirrhosis [5].

Chronic and acute (binge) drinking is a global health and economic problem and a major risk factor for the development of alcoholic liver disease (ALD). The metabolic process for alcohols is mainly carried out through the oxidation pathway by three enzyme systems: ADH, cytochrome P450 2E1 (CYP2E1), and catalase (CAT). The metabolism of alcohol induces CYP2E1, aldehyde oxidase, etc., and induces oxidative stress in the body by producing reactive oxygen species (ROS) [7]. This stress induces an immune response or an inflammatory reaction in various cells in the liver, causing liver damage and disrupting the balance of the antioxidant defense system, leading to various diseases. Therefore, ALD treatment uses compounds or extracts with antioxidant properties, such as vitamin E and phenol compounds [8].

We selected *Zizania latifolia* (*Z. latifolia*) by searching for natural products with high antioxidant activity. The common names for *Z. latifolia* are water bamboo and manchurian wild rice. It has been used in traditional Chinese medicine to treat diabetes and gastrointestinal diseases. In particular, the biological activity and health-promoting effects of *Z. latifolia* are widely recognized in China and South Korea [9]. The antioxidant, wrinkle improvement, anti-inflammatory [10], antiallergic [9], anti-hyperlipidemic [10], immune regulatory [11], and skin protective [12] properties of *Z. latifolia* have recently been studied, but research on the improvement of alcoholic fatty liver using *Z. latifolia* has not yet been performed. Using a binge animal model, we investigated the ability of *Z. latifolia* to ameliorate alcoholic liver disease.

## 2. Material and Methods

### 2.1. Enzyme-Treated Zizania latifolia Extract (ETZL) Preparation

ETZL (DermaNiA™, lot number. BZI30-1910-01), the test material, was provided by BTC Corporation (Sangnok-gu, Ansan, Korea) [12]. Briefly, the dried leaves of *Z. latifolia* were extracted and bioconverted with a mixed hydrolysis enzyme. The enzyme mixture was then inactivated by heating. The supernatant solution of the enzyme extract was acquired with filtering and the residue was re-extracted with water and ethanol (Duksan Science, Seoul, Korea). The filtered residual extract was blended well with the first acquired extract, and the total extract was concentrated and dried to produce ETZL. We confirmed batch–batch variability by measuring tricin, a marker compound for each three batches of ETZL. After that, one batch in which batch–batch variability was confirmed was used for all experiments.

### 2.2. Determination of Total Phenolic Content (TFC)

TFC was measured according to previously described methods [13]. Briefly, 15 µL of ETZL solution (2 mg/mL) was transferred into a 96-well plate in triplicate, mixed with 15 µL of diluted 2 N Folin-Ciocalteu phenol reagent, allowed to react for 8 min in a dark room, and then 30 µL of 0.5 M sodium carbonate was added and it was incubated for 30 min at 37 °C. After incubation, we measured absorbance at 765 nm using a UV/Vis Microplate reader (Epoch 2, Biotek Instruments, Winooski, VT, USA).

### 2.3. High Performance Liquid Chromatography (HPLC) Analysis

**Figure 1:**
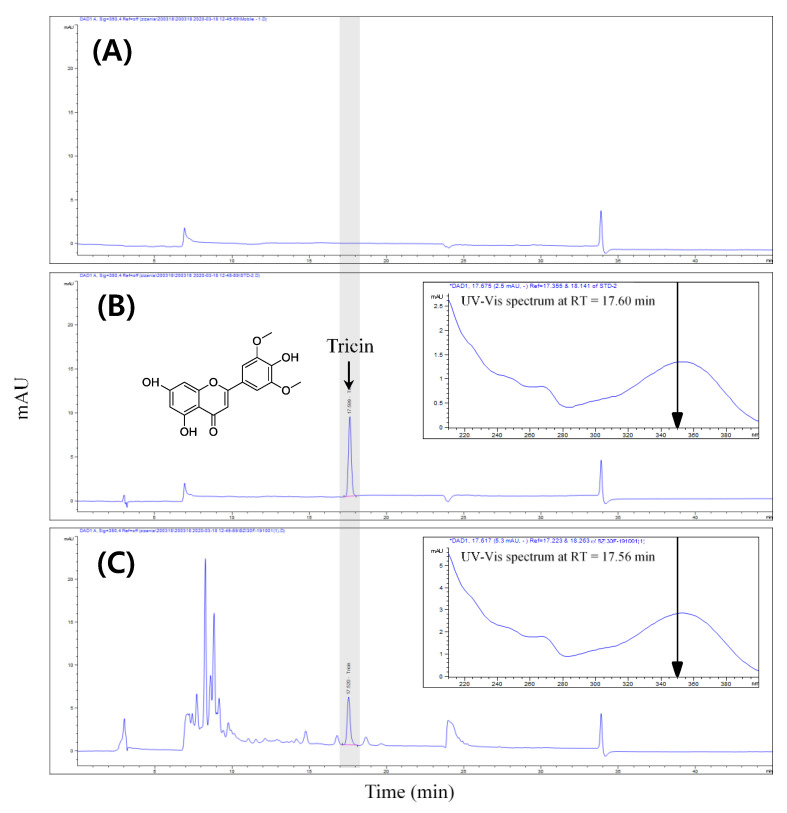
HPLC chromatographic profiles of (**A**) blank, (**B**) tricin standard, and (**C**) enzyme-treated *Z. latifolia* extract (ETZL) with excitation at 350 nm.

The content of tricin in the ETZL was determined by HPLC using an Agilent Infinity 1200 series system with a diode array detector (Agilent Technologies, Palo Alto, CA, USA). The separation of tricin was conducted at 30 °C using a SUPELCO Discovery^®^ C18 column (4.6 × 250 mm, 5 μm, Merck KGaA, Darmstadt, Germany). The mobile phase composition was as follows: A, 0.15% phosphoric acid in water; B, methanol. The gradient elution conditions were as follows: 0–3 min, 20% B; 3–8 min, 20–50% B; 8–20 min, 50–55% B; 20–30 min, 55–85% B; 30–45 min, 85–20% B. The flow rate was 1.0 mL/min and the injection volume was 10 μL. The chromatograms were obtained at 350 nm and UV-Vis spectra were acquired in 190–400 nm (Figure 1). The content of tricin was 1.09 ± 0.01 mg/g in the ETZL.

### 2.4. Non-Cellular Antioxidant Test

The hydroxyl radical scavenging assay was performed using the method described by Klouwen [14]. DPPH (2,2-diphenyl-1-pycryl-hydrazyl) was dissolved in MeOH to prepare a DPPH solution. After mixing the sample solution 1:1 with the prepared DPPH solution, it was shielded from light and left for 30 min at room temperature, and the absorbance was measured at 520 nm. A total of 50 μM ascorbic acid was used as a positive control. The inhibition rate was calculated as follows: [(A1 − A2)/(A0 − A2)] × 100%, where A0 is the absorbance of the control, A1 is the absorbance of the sample, and A2 is the absorbance of the blank sample. Vitamin C (50 μM) was used as a positive control.

### 2.5. Experimental Animals and Design

This study was approved by the Wonkwang University Animal Care Committee (WKU19-42). Male Sprague Dawley rats (6 weeks, weighing 160–170 g) were supplied by Orient Bio (Kwangju, Korea) and were fed laboratory chow (Envigo, Indianapolis, NJ, USA) with access to tap water ad libitum. Rats were acclimatized to temperature (22 ± 2 °C) and humidity (55 ± 5%) controlled rooms with a 12 h light/dark cycle for one week prior to use. The rats were divided into six groups (*n* = 10 per group).

Alcohol diluted with normal saline was administered intragastrically to rats at a dose of 5 g/kg every 12 h for a total of 3 doses. ETZL was orally administered at 100, 200, or 400 mg/kg, 30 min before alcohol administration. Control animals received an identical volume of the vehicle. The positive control was gavaged with silymarin (50 mg/kg). All animals were sacrificed 9 h after the final ethanol administration. Blood was collected from the abdominal aorta using a syringe treated with heparin after anesthesia using ether, and the liver tissue was collected. The collected blood was centrifuged at 3000 rpm for 15 min at 4 °C, and the serum was separated. Serum and liver tissues were stored at −80 °C until analysis.

### 2.6. ALT and AST

ALT and AST levels were measured using the method described by Reitman and Frankel (1957) [15]. Serum and substrate solution were left at 37 ℃ for 30 min and 60 min. Next, 4-DNPH was added and the mixture was left at room temperature for 20 min, followed by a reaction with 0.4 NaOH for 30 min. After the reaction, absorbance was measured at a wavelength of 520 nm.

### 2.7. Triglyceride

The extracted liver tissue was placed in a potassium chloride buffer (pH 7.4) and pulverized for approximately 1 min, and then, 10% liver homogenate was prepared using a triglyceride (TG) kit (Cat no. K622, Biovision, Milpitas, CA, USA), using the Trinder method. Absorbance was measured at 570 nm by combining the assay buffer, probe, and enzyme mix provided in the kit with the liver homogenate.

### 2.8. Histopathlogy

The liver was fixed in 4% formalin followed by 30% sucrose for histological analysis. After the tissue was cryo-embedded, 5 µm thickness cryosections were cut at −15 °C. The sections were stained with Oil red O/hematoxylin and visualized under a microscope (Leica Microsystems, Wetzlar, Germany).

### 2.9. Malondialdehyde (MDA)

Malondialdehyde (MDA) was measured by the Ohkawa (1979) method using the thiobarbituric acid reaction [16]. Using tetraethoxypropane as a standard solution, SDS, thiobarbituric acid, acetic acid, and water were added to the liver homogeneous solution, reacted at 95 °C for 1 h, cooled with ice, and mixed with n-butanol. After this, the sample was centrifuged at 3000 rpm for 10 min, and the supernatant was measured at 532 nm.

### 2.10. Glutathione

Glutathione (GSH) was measured using the Griffith (1979) method [17]. HClO_4_–EDTA solution was added to liver tissue at 5 times the volume and pulverized to make a 20% homogenate. After centrifuging the homogenate at 5000 rpm for 5 min, the supernatant was collected, and the glutathione concentration was diluted within the range of the standard calibration curve using phosphate buffer and used as a sample. Nicotinamide adenine dinucleotide phosphate (NADPH) was added to the microcuvette, sample, or glutathione standard solution containing 5,5′-dithiobis-(2-Nitrobenzoic acid) (DTNB) solution, mixed, and allowed to stand at room temperature for 4 min. Glutathione reductase (GR) was then added and the change in absorbance was measured at 412 nm for approximately 1 min to obtain the slope change.

### 2.11. Cell Culture and Viability Assay

Human liver-derived HepG2 cells were obtained from the American Type Culture Collection (ATCC; Manassas, VA, USA). They were cultured in 24-well culture plates at 2.5 × 10^4^ cells/mL and were pretreated in the presence or absence of *Z. latifolia* at a concentration of 10–300 μg/mL, or silymarin (50 μg/mL) as a positive control for 12 h. Cells were then treated with *t*-BHP for 12 h. Silymarin (50 μg/mL) was used as a positive control. To determine cell viability, cells were added to 200 μL of 1 mg/mL 3-[4,5-dimethylthiazol-2-yl]-2,5-diphenyltetrazolium bromide (MTT; Sigma, St. Louis, MO, USA) solution/well and then incubated for 2 h in a humidified atmosphere (37 °C in 5% CO_2_). The medium was replaced with 200 μL dimethyl sulfoxide (DMSO) and absorbance was measured at 540 nm using a microplate reader (Molecular Devices Inc., Sunnyvale, CA, USA). Cell proliferation was expressed as percentage values in comparison with the negative PBS control, which was considered to represent 100% cell proliferation.

### 2.12. Measurement of ROS Generation

Cells were pretreated in the presence or absence of ETZL at concentrations of 10–300 μg/mL or silymarin (50 μg/mL) as a positive control for 12 h. Next, the cells were treated with *t*-BHP for 12 h. After washing with PBS, the cells were stained with 10 μM 2,7-dichlorofluorescein diacetate in Hank’s balanced salt solution for 30 min in the dark. Subsequently, the cells were washed twice with PBS and lysed with 1% Triton X-100 in PBS at 37 °C for 10 min. Fluorescence was measured at an excitation wavelength of 490 nm and an emission wavelength of 525 nm (Spectramax Gemini XS; Molecular Devices, Sunnyvale, CA, USA). Silymarin (50 μg/mL) was used as a positive control.

### 2.13. Nrf2, HO-1 Protein Expression Analysis

HepG2 cells (2 × 10^6^ cells/mL in 6-well plates) were harvested and pelleted at 200× *g* for 3 min, washed with Tris-buffered saline (TBS; 20 mM Tris, pH 7.5, 130 mM NaCl) containing protease inhibitor and phosphatase inhibitor cocktails, and placed on ice as quickly as possible. The cells were then lysed by direct application of lysis buffer to the dish. A nuclear/cytosol fractionation kit (Bio Vision Technology Inc., New Minas, NS, Canada) was used to separate nuclear and cytoplasmic proteins according to the manufacturer’s protocol. After isolation of the proteins, the concentration of the samples was determined using a micro BCA assay kit (Thermo Fisher Scientific Inc., Waltham, MA, USA). A total of 20 µg of sample per lane was electrophoresed on a 12% reducing SDS–PAGE gel and transferred onto a nitrocellulose membrane, which was blocked with 5% skim milk and sequentially incubated with anti-Nrf2, anti-HO-1, anti-NQO1, anti- GCLC, or anti-GAPDH antibodies at 4 °C overnight (all antibodies were used at a 1:1000 dilution and purchased from Cell Signaling, Danvers, MA, USA). Immunoreactive bands were visualized using horseradish peroxidase-conjugated secondary antibodies (1:1000 dilution; Enzo Life Sciences), followed by ECL detection (Amersham Pharmacia Biotech, Piscataway, NJ, USA). Protein images were captured using a FluorChem E imaging system (ProteinSimple; Santa Clara, CA, USA).

### 2.14. Immunofluorescence

HepG2 cells were grown on 4-well cover slips at a density of 1 × 10^4^ cells/well, and treated with ETZL 100 µg/mL for 12 h at 37 °C. Adherent cells were fixed to slides with paraformaldehyde for 15 min and incubated with 0.3% Trion-X-100 for 10 min at RT. Nonspecific binding was blocked by 3% BSA for 30 min at 37 °C. Immunostaining was performed with an anti-Nrf2 antibody overnight at 4 °C, followed by incubation with a cyanine-3 (Cy3)-conjugated secondary antibody for 2 h at 37 °C. Nuclear staining was performed by incubating with DAPI for 10 min at 37 °C. Increased Nrf2 expression was observed under an inverted microscope (Nikon Corporation, Tokyo, Japan).

### 2.15. Gene Expression Analysis

Total RNA was extracted from harvested cells or hepatic tissue for reverse transcription polymerase chain reaction (RT-PCR) using Easy Blue. The RNA isolation protocol included DNase I treatment. RNA samples were quantified using the standard absorbance OD260 method. Each reaction mix and reaction contained 50–100 ng of RNA and was performed in a total reaction volume of 25 μL. Primer and probe concentrations were 300 and 200 nM, respectively. The conditions for real-time quantitative RT-PCR were as follows: 30 min at 48 °C (RT), 10 min at 95 °C (RT inactivation and initial activation), followed by 40 cycles of amplification for 15 s at 95 °C (denaturation) and 1 min at 60 °C (annealing and extension). The primers and probes for HO-1 (Hs01110250_m1), NQO1 (Hs05639726_s1), GCLC (Hs00155249_m1), FASN (Mm00662319_m1), SREBP1 (Mm00550338_m1), and GAPDH (Hs02758991_g1, Mm99999915_g1) quantitative amplification were validated using the TaqMan Gene Expression Assay (Applied Biosystems). Data analysis was performed using SDS 2.1.1. To normalize the GAPDH housekeeping gene expression, the mathematical model of the relative expression ratio, including PCR efficiency, was chosen and applied for sample quantification.

### 2.16. Statistical Analysis

Data are expressed as the mean ± SD. Significant differences were compared using Student’s *t*-test. Statistical significance was defined as *p* < 0.05. All statistical analyses were performed using GraphPad Prism v.5.0 (Chicago, IL, USA).

## 3. Results

### 3.1. Total Phenolic Content (TFC) in ETZL

Total phenolic content was measured using a Folin–Ciocalteu assay in ETZL. The results were derived from a calibration curve (*y* = 0.04062*x* + 0.0375, *R^2^* = 0.9998) of gallic acid (0–40 mg/mL) and expressed in gallic acid equivalents (GAE) of ETZL per gram. The content of phenolic compounds in ETZL was 4.77 ± 0.08 mg GAE/g.

### 3.2. Effects of ETZL on Hydroxyl Radical Activity

**Figure 2:**
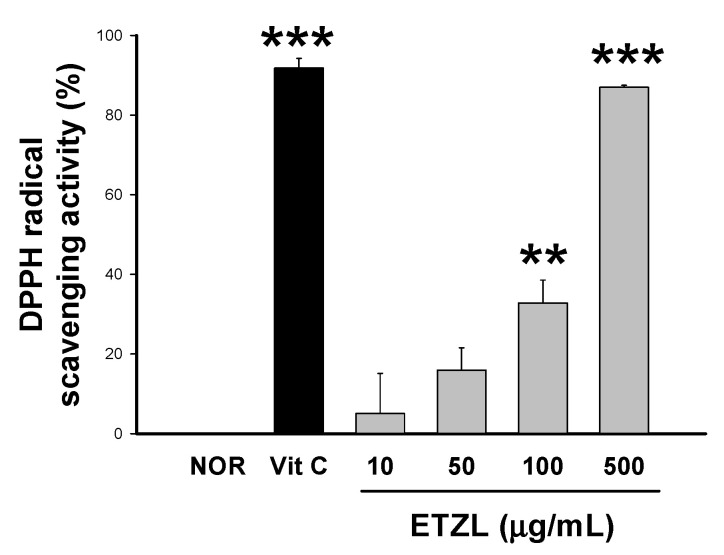
DPPH radical scavenging activity of ETZL. Vitamin C was used as a positive control. Values are expressed as mean ± SD (*n* = 3). Significant differences compared with the normal (NOR) are indicated by ** *p* < 0.01, *** *p* < 0.001.

ETZL was treated with 10, 50, 100, 500 μg/mL, and 50 μM ascorbic acid (vitamin C) as a positive control. Ascorbic acid showed a 91.8% hydroxyl radical scavenging activity compared to the NOR (normal) group. For ETZL, hydroxyl radical scavenging activity increased in a concentration-dependent manner, showing significant increases of 32.8 ± 5.7 and 86.9 ± 0.5% at 100 and 500 μg/mL, respectively (Figure 2).

### 3.3. Effects of ETZL on Liver Function

**Figure 3:**
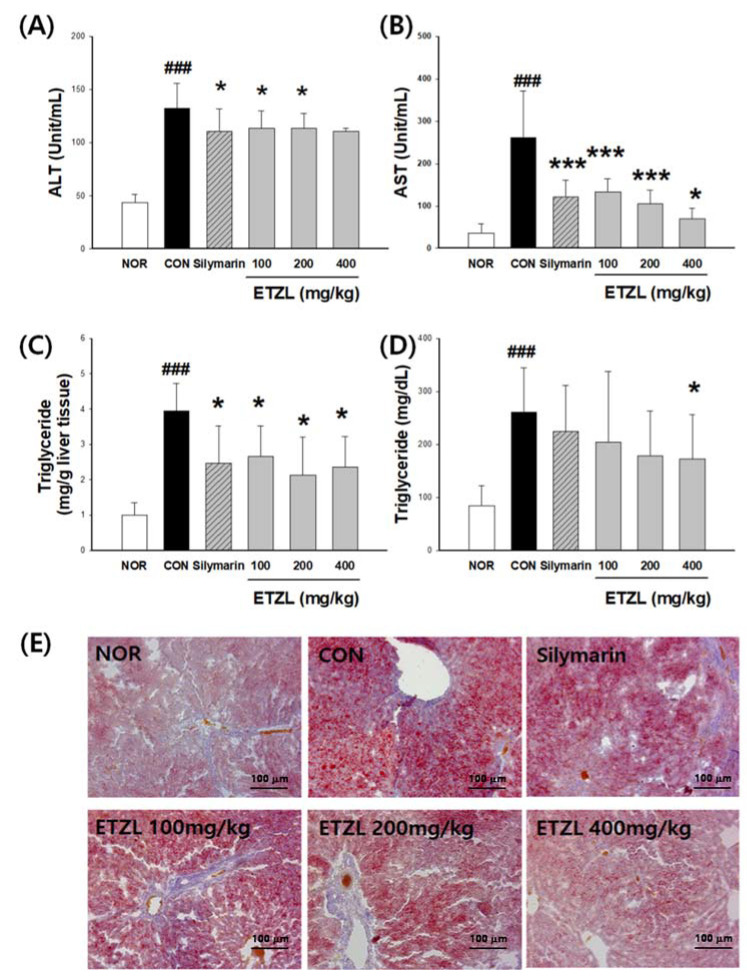
Effects of ETZL treatment on hepatic steatosis in binge rats. SD rats (male, 7 weeks, *n* = 10 rats per group) were used. Rats in the alcohol group were administered 5 g/kg alcohol intragastrically every 12 h for a total of 3 doses. ETZL was orally administered at 100, 200, or 400 mg/kg, 30 min before alcohol administration. Control animals received an identical volume of the vehicle. The positive control was gavaged with silymarin (50 mg/kg). Serum levels of (**A**) ALT, (**B**) AST, (**C**) hepatic triglyceride (TG), (**D**) serum triglyceride (TG), and (**E**) Oil Red O staining of liver sections of rats with ETZL. Values are expressed as mean ± SD. Significant differences compared with the normal (NOR) are indicated by ^###^ *p* < 0.001. Significant differences compared with the control (CON) are indicated by * *p* < 0.05 and *** *p* < 0.001.

To confirm the effect of the administration of ETZL on liver function in an alcoholic fatty liver induction animal model, the liver function indicator enzymes ALT and AST were measured. The positive control (50 mg/kg silymarin) group (ALT: 110.8 ± 20.9 unit/mL, AST: 121.5 ± 39.6 unit/mL) showed an improvement in liver function. When compared to the CON (control) group (ALT: 132.0 ± 23.9 unit/mL), the group administered ETZL at 100, 200, or 400 mg/kg showed ALT measurements of 113.7 ± 16.1, 113.2 ± 14.1, and 110.8 ± 2.7 unit/mL, representing a 13.8%, 14.2%, and 16.0% decrease, respectively. Among these, the 100 and 200 mg/kg groups showed significant differences. For AST, the groups administered ETZL at concentrations of 100, 200, or 400 mg/kg compared to the CON group (AST: 261.0 ± 110.4 unit/mL) showed measurements of 133.6 ± 31.2, 105.5 ± 32.8, and 70.3 ± 24.3 unit/mL, representing significant decreases of 48.8%, 59.6%, and 73.1%, respectively (Figure 3A,B).

### 3.4. Effects of ETZL on Lipid Profiles

Serum TG level was significantly (*p* < 0.01) increased and higher in the CON group (202.0 ± 47.4 mg/dL) than in the NOR group (45.0 ± 14.9 mg/dL). However, compared with the CON group, serum TG levels significantly (*p* < 0.01) decreased in the silymarin (117.5 ± 70.9 mg/dL) and ETZL groups in a dose-dependent manner. In the group administered ETZL at 100, 200, or 400 mg/kg, triglyceride levels were significantly reduced to 40.5%, 51.1%, and 49.7%, respectively, compared to the CON group (Figure 3B).

Figure 3D shows that hepatic TG concentration was 4.4 ± 2.0 mg/g liver, which was higher (*p* < 0.01) in the CON group than in the NOR (1.0 ± 0.2 mg/g liver). During the experimental period, hepatic TG levels decreased in the ETZL-treated groups in a dose-dependent manner, they and were lower than the respective levels in the CON group.

Hepatic lipid accumulation was assessed by Oil Red O staining of triglycerides. The CON group showed a significant accumulation of lipid droplets in the liver. However, this effect was suppressed by ETZL and silymarin. In particular, ETZL at concentrations of 200 and 400 mg/kg was significantly effective in suppressing lipid accumulation (Figure 3E).

### 3.5. Effects of ETZL on Hepatic MDA & GSH

**Figure 4:**
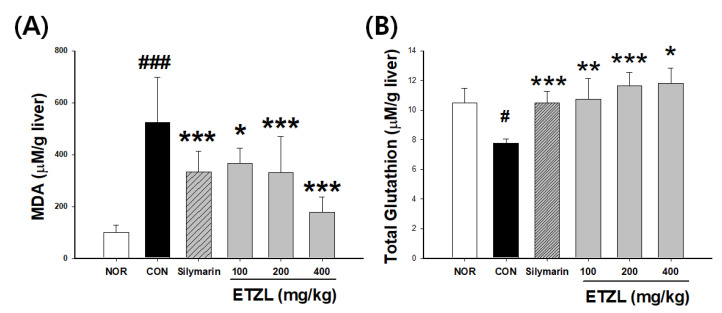
Effect of ETZL on (**A**) MDA and (**B**) GSH levels in liver tissue of binge rats. SD rats (male, 7 weeks, *n* = 10 rats per group) were used. Rats in the alcohol group were administered 5 g/kg alcohol intragastrically every 12 h for a total of 3 doses. ETZL was orally administered at 100, 200, or 400 mg/kg, 30 min before alcohol administration. Control animals received an identical volume of the vehicle. The positive control was gavaged with silymarin (50 mg/kg). Then, hepatic MDA and GSH were measured. Values are expressed as mean ± SD. Significant differences compared with the normal (NOR) are indicated by ^#^ *p* < 0.05, ^###^ *p* < 0.001. Significant differences compared with the control (CON) are indicated by * *p* < 0.05, ** *p* < 0.01 and *** *p* < 0.001.

As shown in Figure 4A, hepatic MDA levels increased (5.2-fold) in the CON group compared to the NOR group. ETZL protected against ethanol-induced liver damage by inhibiting hepatic MDA production: the 100, 200, and 400 mg/kg doses of ETZL inhibited hepatic MDA production by 30.2%, 36.7%, and 66.0%, respectively. Figure 4B shows that hepatic total glutathione decreased (26.1%) in the CON group (7.7 ± 0.3 μM/g liver) compared to the NOR group (10.5 ± 1.0 μM/g liver). ETZL protected against ethanol-induced liver damage by inhibiting total glutathione production: the 100 mg/kg, 200 mg/kg, and 400 mg/kg doses of ETZL inhibited total glutathione production by 38.6%, 50.1%, and 52.3%, respectively.

### 3.6. Effects of ETZL on Cell Viability and ROS Production

**Figure 5:**
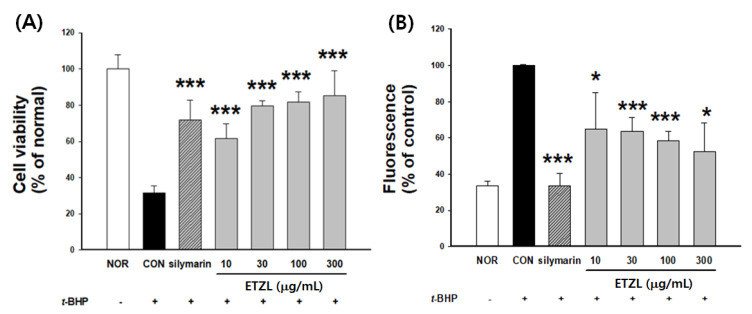
Effects of ETZL on (**A**) cytotoxicity and (**B**) inhibition of reactive oxygen species (ROS) generation in *tert*-butyl hydroperoxide (*t*-BHP)-treated cells. Cells were treated with ETZL and subsequently incubated for 12 h with *t*-BHP (50 µM). The group treated with *t*-BHP was marked with a (+) mark on the X-axis. Cell viability and ROS generation were determined as described in the Materials and Methods section. Silymarin was used as a positive control. Values are expressed as mean ± SD (*n* = 3). Significant differences compared with the *t*-BHP-treated group(CON;control) are indicated by * *p* < 0.05, *** *p* < 0.001.

*Z. latifolia* Turcz was found to be nontoxic up to a concentration of 300 μg/mL, and the rate of ROS production increased by 68.4% in the *t*-BHP-induced group. However, in the group treated with *Z. latifolia* Turcz, the rate of ROS production decreased in a concentration-dependent manner, and there was a significant difference in cell viability (Figure 5).

### 3.7. Effects of ETZL on the NRF2 Pathway

**Figure 6:**
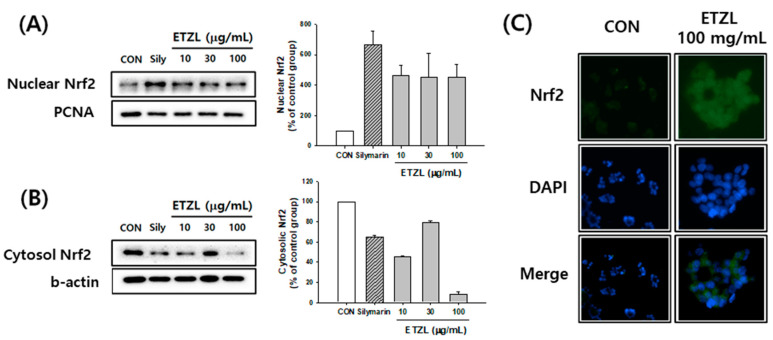
Effect of ETZL on nuclear factor erythroid-derived 2-related factor 2 (Nrf2) activation in HepG2 cells. Cells were treated with indicated concentrations of ETZL for 12 h and Nrf2 translocation was determined. (**A**) ETZL treatment induced Nrf2 protein expression in nucleus. (**B**) ETZL treatment induced Nrf2 protein expression in cytosol. (**C**) ETZL treatment promoted Nrf2 protein nucleus accumulation in HepG2 cells (400×). Values are expressed as mean ± SD (*n* = 3).

**Figure 7:**
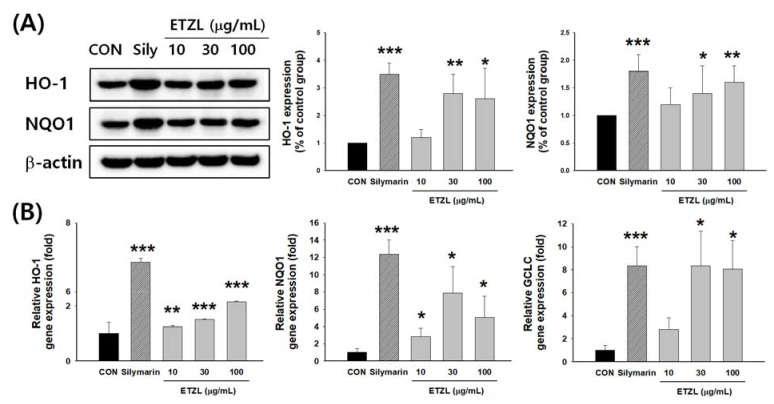
Effects of ETZL on downstream target gene of Nrf2 in hepG2 cells: (**A**) Expression of HO-1 and NQO1 protein. (**B**) Expression of HO-1, NQO1, and GCLC mRNA in HepG2 cells. Cells were incubated for 12 h with the indicated concentrations of ETZL. Values are expressed as mean ± SD (*n* = 3). Significant differences compared with the control (CON) are indicated by * *p* < 0.05, ** *p* < 0.01 and *** *p* < 0.001.

It was confirmed that ETZL increased the protein content of NRF2 in the nucleus and decreased NRF2 in the cytoplasm in a concentration-dependent manner (Figure 6A,B). Immunofluorescence analysis with an anti-Nrf2 antibody revealed that the protein expression of Nrf2 increased with the ETZL concentration (Figure 6C). The expression of HO-1 by ETZL tended to increase in a concentration-dependent manner. Similarly, the protein and mRNA expression of NQO1 tended to increase. In addition, ETZL treatment promoted the expression of glutamate cysteine ligase catalytic subunit (GCLC), a downstream target gene of Nrf2 (Figure 7).

### 3.8. Effects of ETZL on the NRF2 Pathway

**Figure 8:**
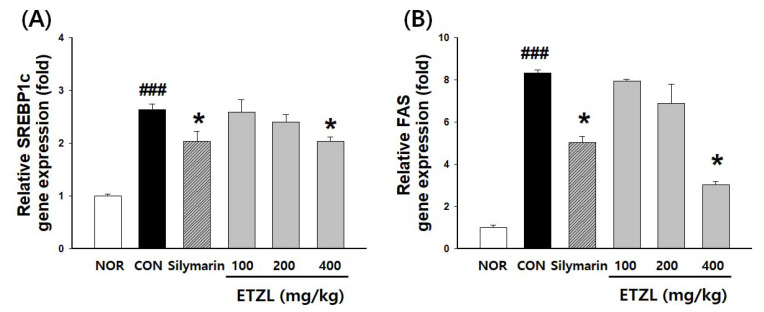
Effects of ETZL on mRNA of lipogenesis related genes in liver tissue of binge rats. mRNA expression level of: (**A**) SREBP-1c, (**B**) FAS in the liver. SD rats (male, 7 weeks, *n*= 3 rats per group) were used. Rats in the alcohol group were administered 5 g/kg alcohol intragastrically every 12 h for a total of 3 doses. ETZL was orally administered at 100, 200, or 400 mg/kg, 30 min before alcohol administration. Control animals received an identical volume of the vehicle. The positive control was gavaged with silymarin (50 mg/kg). Then, lipogenesis-related genes were measured. Values are expressed as mean ± SD. Significant differences compared with the normal (NOR) are indicated by ^###^ *p* < 0.001. Significant differences compared with the control (CON) are indicated by * *p* < 0.05.

SREBP-1c and FAS mRNA levels were significantly increased in the livers of the CON group compared to the NOR group. mRNA expression of SREBP-1c and FAS was significantly lower in the ETZL than in the CON group (Figure 8). These results indicate that the therapeutic effects of ETZL on binge rats are associated with the downregulation of lipogenesis.

## 4. Discussion

*Zizania latifolia* Turcz, a perennial herb belonging to the Graamineae family, grows in groups along rivers and is also called wild rice. The seeds and stems of *Zizania latifolia* Turcz have long been used in the private sector for food and medicinal purposes as a substitute for rice or in the form of tea [9,18]. The leaf of *Z. latifolia* contains proteins, fat, starch, total soluble sugars, ascorbic acid, and polyphenols.

According to a previous study, large amounts of proanthocyanidin [19], total flavonoid content (TFC), and total phenolic content (TPC) [13] were found in the extract of *Z. latifolia*, and a significant antioxidant effect has been reported. Based on these findings, we selected *Z. latifolia* as a candidate for ameliorating alcoholic fatty liver disease. Qian reported that the methanolic extracts of the stems and leaves of *Z. latifolia* showed antioxidant activity against DPPH, with IC50 values of 19.28 and 21.22 mg/mL, respectively [20]. In the present study, the IC50 value for antioxidant activity against DPPH was 0.261 mg/mL. *Z. latifolia* extract showed a higher antioxidant effect at lower concentrations than Qian’s *Z. latifolia* extract [20,21]. Lee et al. reported that, for the first time, one new flavonolignan, one flavone, and three flavonolignans were isolated from the ethylacetate fraction of the aerial parts of *Z. latifolia* as active constituents. Additionally, tricin and five tricin derivatives were reported [22]. Moon et al. reported on the efficacy of enzyme-treated *Zizania latifolia* (ETZL) and its major active compound, tricin, on skin photoaging in SKH-1 hairless mice [12]. The antioxidant effect of tricin has already been verified [23]. Therefore, tricin is expected to be one of a variety of substances in the ETZL extract that shows hepatoprotection through its antioxidative effects. In our previous study, it was confirmed that the content of tricin increased when *Z. latifolia* extract was treated with the enzyme [12]. However, potential synergy effects occur in plant extracts due to the complex mixture of various phytochemicals [24,25,26]. Further research is needed on the active compounds excluding tricin in ETZL.

The application of antioxidants is a rational therapeutic strategy for the prevention and treatment of liver diseases associated with oxidative stress. Oxidative stress is associated with the onset and progression of cirrhosis. Many risk factors, such as the ingestion of alcohol, drugs, and environmental pollutants, can induce oxidative stress in the liver, resulting in serious liver diseases such as alcoholic liver disease and non-alcoholic steatohepatitis [27,28]. Although the conclusions drawn from clinical studies remain uncertain, animal studies have revealed promising in vivo therapeutic effects of antioxidants on liver disease. Natural antioxidants found in edible and medicinal plants often have strong antioxidant, free radical scavenging, and anti-inflammatory properties [8]. Symptoms of liver damage from acute (binge) and chronic alcohol abuse are displayed in a variety of ways, including fatty liver. Therefore, the experimental model was also treated with binge ethanol alone, or with a combination of a high-fat diet or endotoxin with ethanol. 

The binge ethanol-only model used 4–7 g/kg of ethanol at a concentration of 5 g/kg three times at 12 h intervals [29,30,31,32]. A single ethanol binge-only model was chosen to confirm the ameliorating effect of ETZL on fatty liver. ETZL was administered 30 min before ethanol, with a total of three doses. Kirpich et al. reported that three EtOH binges (4.5 g/kg) at 12 h intervals resulted in markedly increased plasma ALT activity, hepatocyte apoptosis, and microvesicular liver steatosis in mice [31]. The activities of ALT and AST increased in livers with alcohol-induced acute hepatotoxicity [6,7]. In this study, it is suggested that the CON group developed hepatotoxicity due to alcohol consumption. On the other hand, the ALT and AST levels of the ETZL group tended to decrease compared to those in the CON group, possibly demonstrating the hepatoprotective effect of ETZL.

Several biochemical mechanisms are involved in the induction of fatty liver and liver damage caused by alcohol consumption, including an increase in intracellular reactive oxygen species (ROS) that affects mitochondrial damage and lipid peroxidation [33,34]. As a result, it is known that alcohol consumption damages cell membranes. The acetaldehyde formed at this time inhibits the release of intracellular substances from the cell by binding to tubulin, and the accumulated proteins, lipids, moisture, and cytoplasm in the cell cause hepatocyte expansion. This has been reported to be characteristic of typical liver damage.

Lipid droplets (LDs) are organelles that maintain lipid homeostasis by storing neutral lipids in the form of triglycerides and cholesterol when energy is excessive, and they act as reservoirs when energy is deficient [35,36]. According to Nguyen et al., DGAT1-dependent LD biogenesis prevents acylcarnitine-induced mitochondrial dysfunction during starvation. LDs can play cytoprotective roles by sequestering FA and reducing the accumulation of various cytotoxic lipid species. Numerous pathways contribute to the synthesis and degradation of hepatic lipid droplets (LDs) [37].

However, abnormal lipid accumulation occurs (also called hepatic steatosis) in LDs of the liver in situations of excessive alcohol consumption or metabolic disorders, such as obesity, diabetes, non-alcoholic fatty liver disease (NAFLD), etc. [38]. It was confirmed that the accumulation of fat in hepatic tissue decreased in the ETZL group in a dependent manner compared with the group treated using the acute binge model. According to previous wild rice (*Zizania latifolia* (Griseb) Turcz) studies, lipid metabolism genes such as SREBP-1c, FASN, ACC, and PPAR were regulated in an NAFLD model [39,40]. Regulation of SREBP1c at the transcriptional level appears to be mediated by one of the metabolites of alcohol metabolism, acetaldehyde [41]. It was confirmed that ETZL significantly reduced SREBP-1c and FAS genes in a hepatic binge model. Taking these results together, it is thought that ETZL represents a mechanism for preventing lipid accumulation in lipid droplets through the inhibition of lipogenesis-related genes.

Reactive oxygen species generated during metabolic processing of ethanol induce oxidative stress in the body because of their high oxidative power and potential to induce certain diseases. GSH is a non-enzymatic part of the antioxidant system and plays an important role in defense by inducing oxidative damage as a thiol in the human body [42]. GSH plays an important role in maintaining the structure and function of normal cells through redox reactions and detoxification. It was expected that the acute binge model would have a reduced GSH compared to the normal group, and that ETZL would contain an antioxidant active ingredient which protected against a decrease in GSH content during alcohol administration. Malondialdehyde (MDA) is a typical active aldehyde produced during lipid peroxidation, and the content of alcohol-induced lipid peroxide in tissues can be measured using the amount of MDA. Compared with the normal group, the alcohol group tended to have significantly increased liver MDA content [43]. However, the administration of ETZL significantly reduced hepatic MDA content. Based on these results, it can be inferred that the alcohol-induced lipid peroxidation process was effectively suppressed by ETZL.

To further study the potential antioxidative stress and antioxidant defense mechanisms of ETZL in oxidative stress liver injury, the expression of ROS, Nrf2, and HO-I in HepG2 cells was investigated. Excessive ROS induces oxidative stress in the liver and suppresses the antioxidant stress defense mechanism, which is defined by abnormal ROS overproduction and is closely associated with many other pathological conditions, including inflammation; this is despite the fact that a strong ROS is essential for maintaining intracellular homeostasis [27,44]. ETZL reduced ROS generated by oxidative stress induction in a concentration-dependent manner.

Recently, studies inducing the expression of antioxidant genes through the activation of Nrf2 using naturally occurring or artificially synthesized small molecules have been conducted [45,46,47]. It was found that Nrf2 has a protective effect against alcohol-induced acute liver injury and carbon tetrachloride-induced liver injury, and that the expression of Nrf2 transcription factor is increased due to oxidative stress by CYP2E1 in hepatocytes [48,49]. Under oxidative stress, oxidative modification of Keap1 allows Nrf2 to release from Keap1, and then Nrf2 translocates into the nucleus. Once in the nucleus, Nrf2 binds the antioxidant response element (ARE) and drives the expression of several downstream genes, such as HO-1, NQO1, and GCLC [42,46,48,50]. The results of the present study showed that the increase in Nrf2 accumulation by ETZL in the nucleus, and the induction of HO-1, NQO1, and GCLC all representative target genes increased the activity of Nrf2 by ETZL. As such, this study is the first to confirm that ETZL administration can alleviate liver injury induced by the alcohol binge inhibition of oxidative stress in rat models. The possible mechanism of ETZL hepatoprotection may be related to its regulation of Nrf2 signaling.

## 5. Conclusions

ETZL demonstrates strong antioxidant activity. ETZL suppressed hepatic lipid accumulation and hepatic damage through Nrf2 activation in a binge drinking model. This study indicates that ETZL could significantly alleviate alcohol-induced hepatotoxicity by increasing anti-oxidative defense. Further investigations, such as animal tests using chronic alcohol models, NAFLD models, as well as human studies, are required to develop hepatoprotective functional foods or pharmaceuticals containing ETZL.

## Data Availability

The data generated during this study are included in this article and are available on request from the corresponding author.

## References

[1] Singh S., Osna N.A., Kharbanda K.K. (2017). Treatment options for alcoholic and non-alcoholic fatty liver disease: A review. World J. Gastroenterol..

[2] Mitra S., De A., Chowdhury A. (2020). Epidemiology of non-alcoholic and alcoholic fatty liver diseases. Transl. Gastroenterol. Hepatol..

[3] Idalsoaga F., Kulkarni A.V., Mousa O.Y., Arrese M., Arab J.P. (2020). Non-alcoholic Fatty Liver Disease and Alcohol-Related Liver Disease: Two Intertwined Entities. Front. Med..

[4] Toshikuni N., Tsutsumi M., Arisawa T. (2014). Clinical differences between alcoholic liver disease and nonalcoholic fatty liver disease. World J. Gastroenterol..

[5] Shoreibah M., Raff E., Bloomer J., Kakati D., Rasheed K., Kuo Y.F., Singal A.K. (2016). Alcoholic liver disease presents at advanced stage and progresses faster compared to non-alcoholic fatty liver diseas. Ann. Hepatol..

[6] Arab J.P., Roblero J.P., Altamirano J., Bessone F., Chaves Araujo R., Higuera-De la Tijera F., Restrepo J.C., Torre A., Urzua A., Simonetto D.A. (2019). Alcohol-related liver disease: Clinical practice guidelines by the Latin American Association for the Study of the Liver (ALEH). Ann. Hepatol..

[7] Louvet A., Mathurin P. (2015). Alcoholic liver disease: Mechanisms of injury and targeted treatment. Nat. Rev. Gastroenterol. Hepatol..

[8] Tu Y., Zhu S., Wang J., Burstein E., Jia D. (2019). Natural compounds in the chemoprevention of alcoholic liver disease. Phytother. Res..

[9] Yu X., Chu M., Chu C., Du Y., Shi J., Liu X., Liu Y., Zhang H., Zhang Z., Yan N. (2020). Wild rice (Zizania spp.): A review of its nutritional constituents, phytochemicals, antioxidant activities, and health-promoting effects. Food Chem..

[10] Zhang H., Cao P., Agellon L.B., Zhai C.K. (2009). Wild rice (*Zizania latifolia* (Griseb) Turcz) improves the serum lipid profile and antioxidant status of rats fed with a high fat/cholesterol diet. Br. J. Nutr..

[11] Moghadasian M.H., Zhao R., Ghazawwi N., Le K., Apea-Bah F.B., Beta T., Shen G.X. (2017). Inhibitory Effects of North American Wild Rice on Monocyte Adhesion and Inflammatory Modulators in Low-Density Lipoprotein Receptor-Knockout Mice. J. Agric. Food Chem..

[12] Moon J.M., Park S.H., Jhee K.H., Yang S.A. (2018). Protection against UVB-Induced Wrinkle Formation in SKH-1 Hairless Mice: Efficacy of Tricin Isolated from Enzyme-Treated *Zizania latifolia* Extract. Molecules.

[13] Chu M.-J., Liu X.-M., Yan N., Wang F.-Z., Du Y.-M., Zhang Z.-F. (2018). Partial Purification, Identification, and Quantitation of Antioxidants from Wild Rice (*Zizania latifolia*). Molecules.

[14] Klouwen H.M. (1962). Determination of the sulfhydryl content of thymus and liver using DPPH. Arch. Biochem. Biophys..

[15] Reitman S., Frankel S. (1957). A colorimetric method for the determination of serum glutamic oxalacetic and glutamic pyruvic transaminases. Am. J. Clin. Pathol..

[16] Ohkawa H., Ohishi N., Yagi K. (1979). Assay for lipid peroxides in animal tissues by thiobarbituric acid reaction. Anal. Biochem..

[17] Griffith O.W., Anderson M.E., Meister A. (1979). Inhibition of glutathione biosynthesis by prothionine sulfoximine (S-n-propyl homocysteine sulfoximine), a selective inhibitor of gamma-glutamylcysteine synthetase. J. Biol. Chem..

[18] Surendiran G., Alsaif M., Kapourchali F.R., Moghadasian M.H. (2014). Nutritional constituents and health benefits of wild rice (*Zizania* spp.). Nutr. Rev..

[19] Chu M.-J., Du Y.-M., Liu X.-M., Yan N., Wang F.-Z., Zhang Z.-F. (2019). Extraction of Proanthocyanidins from Chinese Wild Rice (*Zizania latifolia*) and Analyses of Structural Composition and Potential Bioactivities of Different Fractions. Molecules.

[20] Qian B., Luo Y., Deng Y., Cao L., Yang H., Shen Y., Ping J. (2012). Chemical composition, angiotensin-converting enzyme-inhibitory activity and antioxidant activities of few-flower wild rice (*Zizania latifolia* Turcz.). J. Sci. Food Agric..

[21] Masarone M., Rosato V., Dallio M., Gravina A.G. (2018). Role of Oxidative Stress in Pathophysiology of Nonalcoholic Fatty Liver Disease. Oxidative Med. Cell. Longev..

[22] Lee S.-S., Baek Y.-S., Eun C.-S., Yu M.-H., Baek N.-I., Chung D.-k., Bang M.-H., Yang S.-A. (2015). Tricin derivatives as anti-inflammatory and anti-allergic constituents from the aerial part of *Zizania latifolia*. Biosci. Biotechnol. Biochem..

[23] Duarte-Almeida J.M., Negri G., Salatino A., de Carvalho J.E., Lajolo F.M. (2007). Antiproliferative and antioxidant activities of a tricin acylated glycoside from sugarcane (Saccharum officinarum) juice. Phytochemistry.

[24] Malongane F., McGaw L.J., Mudau F.N. (2017). The synergistic potential of various teas, herbs and therapeutic drugs in health improvement: A review. J. Sci. Food Agric..

[25] Yang Y., Zhang Z., Li S., Ye X., Li X., He K. (2014). Synergy effects of herb extracts: Pharmacokinetics and pharmacodynamic basis. Fitoterapia.

[26] Liu J., Liu J., Shen F., Qin Z., Jiang M., Zhu J., Wang Z., Zhou J., Fu Y., Chen X. (2018). Systems pharmacology analysis of synergy of TCM: An example using saffron formula. Sci. Rep..

[27] Luangmonkong T., Suriguga S., Mutsaers H.A.M., Groothuis G.M.M., Olinga P., Boersema M. (2018). Targeting Oxidative Stress for the Treatment of Liver Fibrosis. Rev. Physiol. Biochem. Pharmacol..

[28] Jorgačević B., Vučević D., Samardžić J., Mladenović D., Vesković M., Vukićević D., Ješić R., Radosavljević T. (2021). The Effect of CB1 Antagonism on Hepatic Oxidative/Nitrosative Stress and Inflammation in Nonalcoholic Fatty Liver Disease. Curr. Med. Chem..

[29] Liu X., Connaghan K.P., Wei Y., Yang Z., Li M.D., Chang S.L. (2016). Involvement of the Hippocampus in Binge Ethanol-Induced Spleen Atrophy in Adolescent Rats. Alcohol. Clin. Exp. Res..

[30] Aroor A.R., Roy L.J., Restrepo R.J., Mooney B.P., Shukla S.D. (2012). A proteomic analysis of liver after ethanol binge in chronically ethanol treated rats. Proteome Sci..

[31] Kirpich I., Ghare S., Zhang J., Gobejishvili L., Kharebava G., Barve S.J., Barker D., Moghe A., McClain C.J., Barve S. (2012). Binge alcohol-induced microvesicular liver steatosis and injury are associated with down-regulation of hepatic Hdac 1, 7, 9, 10, 11 and up-regulation of Hdac 3. Alcohol. Clin. Exp. Res..

[32] Abdelmegeed M.A., Banerjee A., Jang S., Yoo S.H., Yun J.W., Gonzalez F.J., Keshavarzian A., Song B.J. (2013). CYP2E1 potentiates binge alcohol-induced gut leakiness, steatohepatitis, and apoptosis. Free. Radic. Biol. Med..

[33] Donato M., Tolosa L. (2021). High-Content Screening for the Detection of Drug-Induced Oxidative Stress in Liver Cells. Antioxidants.

[34] Arauz J., Ramos-Tovar E., Muriel P. (2016). Redox state and methods to evaluate oxidative stress in liver damage: From bench to bedside. Ann. Hepatol..

[35] Mashek D.G. (2020). Hepatic lipid droplets: A balancing act between energy storage and metabolic dysfunction in NAFLD. Mol. Metab..

[36] Olzmann J.A., Carvalho P. (2019). Dynamics and functions of lipid droplets. Nat. Rev. Mol. Cell Biol..

[37] Nguyen T.B., Louie S.M., Daniele J.R., Tran Q., Dillin A., Zoncu R., Nomura D.K., Olzmann J.A. (2017). DGAT1-Dependent Lipid Droplet Biogenesis Protects Mitochondrial Function during Starvation-Induced Autophagy. Dev. Cell.

[38] Gluchowski N.L., Becuwe M., Walther T.C., Farese R.V. (2017). Lipid droplets and liver disease: From basic biology to clinical implications. Nat. Rev. Gastroenterol. Hepatol..

[39] Han S.-F., Zhang H., Zhai C.-K. (2012). Protective potentials of wild rice (*Zizania latifolia* (Griseb) Turcz) against obesity and lipotoxicity induced by a high-fat/cholesterol diet in rats. Food Chem. Toxicol..

[40] Han S., Zhang H., Qin L., Zhai C. (2013). Effects of dietary carbohydrate replaced with wild rice (*Zizania latifolia* (Griseb) Turcz) on insulin resistance in rats fed with a high-fat/cholesterol diet. Nutrients.

[41] He Q., Diao Y., Zhao T., Hou B., Ngokana L.D., Liang H., Nie J., Tan P., Huang H., Li Y. (2018). SREBP1c mediates the effect of acetaldehyde on Cidea expression in Alcoholic fatty liver Mice. Sci. Rep..

[42] Lu S.C. (2020). Dysregulation of glutathione synthesis in liver disease. Liver Res..

[43] Gao B., Bataller R. (2011). Alcoholic liver disease: Pathogenesis and new therapeutic targets. Gastroenterology.

[44] Prieto I., Monsalve M. (2017). ROS homeostasis, a key determinant in liver ischemic-preconditioning. Redox Biol..

[45] Xu D., Xu M., Jeong S., Qian Y., Wu H., Xia Q., Kong X. (2018). The Role of Nrf2 in Liver Disease: Novel Molecular Mechanisms and Therapeutic Approaches. Front. Pharmacol..

[46] Taguchi K., Kensler T.W. (2020). Nrf2 in liver toxicology. Arch. Pharmacal Res..

[47] Chambel S.S., Santos-Gonçalves A., Duarte T.L. (2015). The Dual Role of Nrf2 in Nonalcoholic Fatty Liver Disease: Regulation of Antioxidant Defenses and Hepatic Lipid Metabolism. BioMed Res. Int..

[48] Rejitha S., Prathibha P., Indira M. (2015). Nrf2-mediated antioxidant response by ethanolic extract of Sida cordifolia provides protection against alcohol-induced oxidative stress in liver by upregulation of glutathione metabolism. Redox Rep. Commun. Free Radic. Res..

[49] Kensler T.W., Wakabayashi N., Biswal S. (2007). Cell survival responses to environmental stresses via the Keap1-Nrf2-ARE pathway. Annu. Rev. Pharmacol. Toxicol..

[50] Cederbaum A.I. (2013). Nrf2 and antioxidant defense against CYP2E1 toxicity. Sub-Cell. Biochem..

